# Prospects for fistula technologies in the study of digestion and metabolism in birds

**DOI:** 10.3389/fphys.2026.1780664

**Published:** 2026-02-26

**Authors:** Vladimir G. Vertiprakhov, Sergei Yu Zaitsev

**Affiliations:** 1 Department of Animal Physiology, Ethology and Biochemistry, Russian State Agrarian University, Moscow, Russia; 2 Moscow Timiryazev Agricultural Academy, Moscow, Russia; 3 Analytical Biochemistry Group, Department of Physiology and Biochemistry of Farm Animals, Federal Research Center for Animal Husbandry Named After Academy Member L. K. Ernst, Moscow, Russia

**Keywords:** fistula method, broiler chickens, digestion, metabolism, physiological control

## 1 Introduction

As part of physiological and biochemical studies of digestive processes, it is advisable to study the mechanisms of action of various dietary components and metabolic processes primarily in animals. Since it is not always possible to use a human or large farm animal model in digestion studies, it is advisable to study the mechanisms of action of various dietary components and metabolism in small or laboratory animals. That is why, dogs, rats, pigs (Bornhorst and Singh, 2014), and chickens (Batoev, 2001; Vertiprakhov et al., 2019; Vertiprakhov, 2022) are commonly used for digestion experiments. In animal digestion studies, chyme samples are collected from the gastrointestinal tract, which can be done using two main methods: cannulation and slaughter. Fistula (*cannula*) placement involves surgically inserting a cannula into the wall of the gastrointestinal tract. The outer end of the *cannula* protrudes beyond the skin and contains a removable stopper that allows for sample extraction (Bornhorst and Singh, 2014). The advantage of cannulation is that it allows for sampling from the same animal over a long period of time. Chronic experiments are a powerful tool for studying digestion in animals, allowing the dynamics of physiological processes to be monitored in real time, as demonstrated in studies of carbohydrate digestion in pigs (Serena et al., 2006; Hasjim et al., 2010). The use of cannulated animals provides unique data on the kinetics of enzymatic digestion, intestinal motility, and microbial fermentation, which is unavailable with static methods such as postmortem analysis.

## 2 Features of the gastrointestinal tract in birds

The digestive system of birds differs from that of mammals (DeGolier et al., 1999; Batoev, 2001; Vertiprakhov et al., 2019; Vertiprakhov, 2022). Birds combine the use of HCl and pepsin with mechanical digestion in the proventriculus and gizzard. Since the pH of the gizzard is acidic, stones with a high CaCO_3_ content dissolve quickly. When birds are fed compound feed, the role of “muscular digestion” is reduced. From the gizzard, the food bolus enters the duodenum, which receives an alkaline secretion from the pancreas and liver (Batoev, 2001; Vertiprakhov et al., 2019; Vertiprakhov, 2022). The entire intestine in birds is shorter than in mammals. The large intestine in birds is relatively short, comprising the cecum, rectum, and cloaca. The cecum in adult chickens is richin lymphoid tissue, indicating its role in intestinal immune responses. The cecum provides a favorable environment for the growth of anaerobic bacteria, which are capable of synthesizing water-soluble vitamins. In the cecum, carbohydrates not exposed to microorganisms in the small intestine are fermented, as in mammals, to form volatile fatty acids. It is supposed that the birds cannot utilize microbial proteins in their caecum. Significant water absorption occurs in the rectum and cloaca. Bird feces typically contain some white uric acid crystals. The cloacal wall contains the “bursa of Fabricius”, an important lymphoid organ. Overall, the bird intestine serves not only as a digestive system but also as a barrier to immune complexes and other factors (Batoev, 2001; Vertiprakhov et al., 2019; Vertiprakhov, 2022).

## 3 Major methods for studying digestion in poultry

**FIGURE 1:**
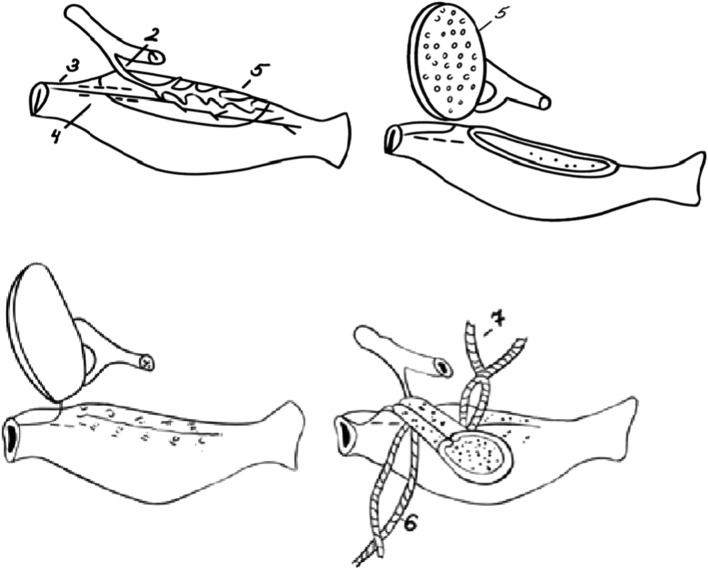
Scheme of the surgical procedure for creating an isolated stomach in a duck (accordin P.P. Berdnikov): 1-coeliac artery; 2-dorsal gastric artery; 3-dorsal branch of the left vagus nerve; 4- area of nerve preparation; 5-gastric wall flap; 6-suture securing the ventricle to the main stomach; 7- purse-string suture (adapted from Batoev, 2001).

Birds are a good model for studying digestion and metabolic regulatory processes, since, along with hormones, pancreatic enzymes act as signaling molecules (Luo et al., 2007; Chia et al., 2011; Bohn et al., 2018; Vertiprakhov et al., 2023). However, surgical insertion of a cannula into the stomach and intestines is considered the most promising method. For example, the problem of obtaining pure gastric juice in a chronic experiment in ducks was successfully solved first by Berdnikov (1990). The author adopted I.P. Pavlov’s classical method of creating an isolated stomach for mammals as a basis, taking into account the morphofunctional characteristics of birds. The surgical procedure involves preserving the innervations of the small ventricle by dissecting a section of the *vagus* nerve in the area of the gastric flap resection, which will later be used to create the isolated ventricle. The gastric wall flap is dissected circumferentially through all layers, including the dissected nerve, and the small ventricle is sutured. The ventricle is supplied with blood via the dorsal gastric artery and innervated by a branch of the vagus nerve. The ventricle is secured to the large ventricle with a suture. A cannula inserted into the isolated ventricle is brought out and secured to the skin with a purse-string suture (Figure 1).

Using this method for obtaining gastric juice in ducks, the patterns of gastric juice secretion and its regulatory mechanisms were studied. The conditions for the adaptation of gastric glands to changes in diet were determined. Data on the functional relationships of the digestive glands were presented (Berdnikov et al., 2012). One method for obtaining pure pancreatic juice in chickens is cannulation of the pancreatic duct (Hulan et al., 1972). A surgical technique for cannulation of the main pancreatic duct in 14-week-old chicks has been developed. A silicone cannula is inserted into the main pancreatic duct, the free end of which is removed through a puncture in the abdominal wall and connected to a collecting tube made of polyethylene 90. The collection tube is then connected to a tube that is placed in a thermos with ice when the operated chick is placed in the cage for fixation. It was found that no special postoperative care was required. The pancreatic secretion was successfully collected within 24 h for 3 months after the catheter was placed. None of the chickens lost weight, and most even gained weight during the experiment. Successful catheter insertion into the pancreatic duct was performed in 90%–95% of cases. A significant disadvantage of this method was the further activation of proteases, since they are activated in the 12 denum. Batoev Ts. Zh. and coworkers developed a fundamentally new method for studying the exocrine function of the pancreas in poultry (Batoev, 2001). It is based on the implantation of pancreatic ducts into an isolated segment of the intestine and the formation of a pancreaticoduodenal anastomosis (Batoev, 2001).

This method was used to study the characteristics of the exocrine function of the pancreas in chickens and ducks.

## 4 Innovative methods for determining the taste and nutritional properties of poultry feed

Since poultry is a convenient model for studying biological processes related to metabolism, new methods for use in physiological experiments are being sought. The surgical procedures proposed by Berdnikov, Batoev and Batoeva (Batoev, 2001; Berdnikov et al., 2012) are difficult to perform on poultry, so simpler and more effective methods are being developed for chronic experiments studying metabolic pathways involving digestive enzymes. This requires inserting a cannula into the duodenum opposite the confluence of the pancreatic and bile ducts (Vertiprakhov, 2022).

Surgical procedures were performed using sedatives and anesthetics. The chicken was fixed in a left lateral position in a special device. An incision was made on the right side of the last rib along the edge of the lateral process of the sternum, 4–5 cm in length. The duodenum was removed, the entry of the ducts into the duodenum was identified, and a 0.5–0.6 cm purse-string suture was placed opposite it. An incision was made within the purse-string suture, a cannula was inserted, and the purse-string suture was tightened (Figure 1). The area around the implanted cannula was carefully prepared, and an additional purse-string suture was placed, if necessary. The intestine was placed deep into the “thoracoabdominal” cavity, and the surgical wound was closed with interrupted sutures, covering all layers. After the surgery, the bird had access to water for 16–18 h, but was not given food. Physiological experiments began in 5–7 days after surgery, when the bird’s health had fully recovered.

To study duodenal digestion and the role of pancreatic enzymes, the authors propose a method that is significantly simpler to implement than the method proposed by (Batoev, 2001). The method involves inserting a cannula into the duodenum opposite the confluence of two bile ducts and three pancreatic ducts (Vertiprakhov, 2022). We have developed a specially designed cannula suitable for laying hens and broiler chickens of various ages (Russian Federation, 2024). By studying duodenal enzyme activity in broiler chickens, the authors concluded that it is possible to determine the taste sensations of feed. Experiments conducted on broiler chickens and hens, using various additives in their diets, confirmed our hypothesis (Sergeenkova and Vertiprakhov, 2024; Sergeenkova et al., 2024a; Sergeenkova et al., 2024b).

Nutrient absorption in the diet receives the greatest attention, as it significantly impacts animal productivity. Birds are prone to ideal fistula formation. One method proposed for obtaining ideal contents in poultry was proposed by Japanese scientists (Isshiki et al., 1989). Unlike the classical method for determining fecal accessibility, such accessibility has its own unique properties (Zhuravlev et al., 2020). Along with fistula technologies for studying digestion, it is necessary to simultaneously determine the absorption of amino acids and di- and tripeptides in the blood and analyze digestive en zymes in the blood (trypsin), which are biomarkers of animal metabolism (Pierzynowska et al., 2025). This provides a comprehensive and more objective approach to assessing the body’s adaptive capabilities and the health of the digestive system.

## 5 Conclusion

It is important to highlight that the use of fistula technologies in the study of animal metabolism has advantages over post-mortem collection of research material. It allows for the collection of contents from a healthy living organism multiple times at regular intervals in a single animal to determine the dynamics of different phases of the secretory process in real time. Modern methods for determining enzymatic activity allow for an objective assessment of the process of adaptation to the arrival of a nutrient substrate in the digestive tract and comparison with the initial period, as well as the availability of amino acids and di-, tripeptides in the blood and the level of metabolism using a biomarker—trypsin activity in the blood in the pre- and postprandial periods. This allows for the determination of regulatory processes in the bird’s body and the mechanism of action of various feed additives, as well as the reactivity of adaptive responses in assessing selection parameters when developing new animal crosses while simultaneously comparing the expression of genes associated with metabolism.
